# Identifying the most suitable treatment for depression based on patients’ attachment: study protocol for a randomized controlled trial of supportive-expressive vs. supportive treatments

**DOI:** 10.1186/s12888-018-1934-1

**Published:** 2018-11-12

**Authors:** Sigal Zilcha-Mano, Tohar Dolev, Liat Leibovich, Jacques P. Barber

**Affiliations:** 1 The Department of Psychology, University of Haifa, Mount Carmel, 31905 Haifa, Israel; 2 The Gorden F. Derner School of Psychology, Adelphi University, New York, USA

**Keywords:** Personalized treatment, Supportive expressive treatment, Alliance, Psychodynamic treatment, Attachment

## Abstract

**Background:**

In the absence of one intervention that can cure all patients with major depressive disorder (MDD), the leading cause of disability worldwide, increased attention has been focused on selecting the best treatment based on patient characteristics. Theory-driven hypotheses for selecting the best treatments have not yet been adequately investigated. The present study tested the *a priory* hypothesis that attachment orientations may determine whether patients benefit more from a treatment where alliance provides a facilitative environment for the treatment to work, as in the case of supportive-expressive psychotherapy, vs. where alliance is conceptualized as an active ingredient in itself, as in the case of supportive psychotherapy.

**Method/design:**

To test the hypothesis that attachment orientation moderates the effect of treatment condition on outcome, we conduct a randomized controlled trial (RCT). One hundred patients are randomized to 16 sessions of either supportive-expressive or supportive psychotherapy for MDD, conducted by experienced psychologists. The primary outcome is change in the Hamilton Rating Scale for Depression. Secondary outcome measures include self-reported depressive and other symptoms, psychological and interpersonal functioning, quality of life, and the presence of the diagnosis of depression. Additional measures include hormonal levels, motion synchrony, and acoustic attributes, performance on cognitive tasks, and narrative material (collected from the sessions and from interviews).

**Discussion:**

The RCT will expand our understanding of how the outcome of treatment can be optimized by identifying the most promising role of alliance in treatment, based on patients’ pre-treatment attachment orientation. Results will contribute to the ongoing theoretical debate concerning the differential efficacy of various psychotherapeutic approaches for patients with different attachment orientations. The RCT will also contribute to progress toward personalized treatment by informing therapists about which of two approaches are most effective with patients based on their attachment styles.

**Trial registration:**

Clinicaltrials.gov Identifier: NCT02728557 submitted on the 15.3.16. Funding: The Israel Science Foundation. Trial status: Recruitment is ongoing.

## Background

Effective psychotherapy is characterized by good working alliance between patient and therapist [1]. The working alliance is commonly defined as the emotional bond established in the therapeutic dyad, and the agreement between the two about the goals of therapy and the tasks necessary to achieve them [2, 3]. The quality of the therapeutic alliance is a consistent predictor of outcome in psychotherapy, with stronger alliances being associated with better therapeutic outcomes [4], even after establishing the temporal precedence between alliance and symptoms [5–7].

Recently, it has been demonstrated that the effect of alliance on outcome is comprised of two main components, trait-like and state-like, each playing a distinct role in treatment [8, 9]. The trait-like component of alliance serves as a precondition for therapeutic work, essential for forming the environment required to provide effective treatment. It is a product of the patients’ (and the dyads’) trait-like characteristics. Some individuals have better trait-like capacity to form strong and satisfying relationships with significant others. Empirical studies suggest that these capabilities affect their tendency to create a strong helping relationship with their therapist [10–13], which is the environment required to conduct any effective treatment. Indeed, patients with such adaptive trait-like characteristics improve more following treatment [14–16]. This component, however, is not sufficient in itself to induce change, and it is mainly a product of other trait-like characteristics of the patient.

By contrast, the state-like component of alliance serves as a mechanism of change in itself, with changes in this component of the alliance being the cause of subsequent symptomatic change [9]. The state-like component represents the role of alliance as an active ingredient, capable of inducing therapeutic change in itself. During such a process of therapeutic change, the patient develops abilities to form a strong and satisfactory alliance with the therapist, resulting in better outcomes. Empirical studies suggest that state-like changes in alliance significantly predict subsequent treatment outcome over the course of treatment [6, 15, 16], supporting their role in bringing about therapeutic change.

In some treatments, such as supportive-expressive (SE) treatment, alliance is perceived as playing a role in creating a facilitative environment for treatment to work. In such instances, the trait-like component of alliance can be said to play a dominant role in treatment. By contrast, others treatments, such as supportive treatment, perceive the alliance as an active ingredient in itself. In such instances, the state-like component of alliance can be said to play a dominant role in treatment. In SE treatment, the active ingredient is the promotion of insight through interpretations focusing on the patients’ core conflictual relationship themes (CCRT). In this treatment, the alliance functions as the helpful collaborative environment needed to effectively interpreted the patients’ CCRT and work them through with the patient [17, 18]. By contrast, in supportive treatment, the alliance with the therapist is perceived as a core active ingredient responsible for the success of treatment, potentially through the formation of a corrective experience [19, 20]. Given the distinct roles of alliance in each of the treatments on the one hand, along with the many commonalities between those treatments on the other hand, the question arises whether one treatment is more effective than the other. Although no study has directly examined this question in the treatment of depression, a recent meta-analysis divided studies into supportive and expressive modes by coding them based on the relative weight they place on either pole [21]. The findings resemble those of the majority of meta-analyses comparing the efficacy of active treatments for depression: no significant differences were found between treatments with greater supportive focus and those with greater expressive focus.

Recent studies suggest that although no significant differences emerge between treatments at the sample level, certain subgroups of patients fare best in one treatment than in the other (for reviews, see [22, 23]). Findings across many types of treatments, especially for major depressive disorder (MDD), demonstrate that in trials that failed to find any differences between treatments at the sample level, certain subgroups of patients with distinct pre-treatment characteristics showed an ability to benefit more from one treatment than from the other (e.g., [24, 25]). Zilcha-Mano and Errázuriz [26] investigated this issue with respect to alliance, seeking to examine whether some patients benefit more from the role of alliance as an active ingredient in itself, while other patients from the role of alliance as enabling other active ingredients to induce change. They found that treatments with a more dominant state-like component of alliance (that is, where alliance is conceived as playing an active role in itself) were more helpful for patients reporting more interpersonal problems than those reporting less interpersonal problems [26]. These findings are promising but preliminary because they are not based on randomizing patients to treatments where the state-like role of alliance is more or less dominant than its trait-like role, but on ordinarily occurring differences between treatments in naturalistic settings. Furthermore, because the study included only a broad measurement of interpersonal problems, based on a subscale of the Outcome Questionnaire (OQ; [27]), it was not possible to infer how treatment may be optimized by tailoring the alliance function to the patients’ interpersonal characteristics and needs. One of the bases for differentiating between individuals’ interpersonal characteristics and needs is attachment theory.

According to attachment theory [28–30], people learn to trust others in times of need through a series of interactions with significant others (attachment figures). These interactions, which begin to accumulate as early as infancy, are encoded in internal representations (working models) of the self and others. Throughout life, these working models influence the individual’s expectations, perceptions, and behaviors in interpersonal relationships. Individual differences in adults’ working models are commonly conceptualized on two orthogonal dimensions of attachment orientation: anxiety and avoidance [31]. Individuals who score high on the anxiety dimension hold expectations of being rejected and disappointed in interpersonal relationships and tend to hyperactivate attachment strategies: energetic attempts to attain greater proximity, support, and love, combined with a lack of confidence that it would be provided. Those who score high on the avoidance dimension hold few positive expectations from others and are characterized by deactivating attachment strategies: inhibition of proximity-seeking tendencies, maintenance of emotional and cognitive distance from others, and compulsive self-reliance. People who score low on both dimensions are said to be securely attached and are thought to possess mental representations of comforting and support-providing attachment figures. Over a hundred studies have established that these two dimensions demonstrate different needs in interpersonal relationships and benefit from different types of help [32].

Recent theories and empirical research suggest a promising role for patients’ attachment orientation in determining which treatment to assign to patients depending on whether the alliance serves as an active ingredient or whether it facilitates the effective implementation of other active ingredients [33–35]. Theoretical conceptualizations suggest that anxiously attached patients, who often employ hyperactivating strategies, may benefit most from counter-complementary deactivating techniques, where alliance plays a trait-like role; in the case of SE psychotherapy, this means facilitating the conditions needed for effective expressive work in treatment. The reverse would be true for avoidantly attached patients, who demonstrate deactivating strategies and may benefit most from counter-complementary hyperactivating techniques, in which alliance plays the role of an active ingredient in itself, such as in supportive treatment, encouraging feelings of intimacy in the patient-therapist relationship. These assumptions are consistent with the theory of opposites, which posits that helpful therapy requires the therapist to behave in a way that is antithetical (“opposite”) to the interpersonal behavior of the patient [36, 37], and with empirical studies supporting its validity in determining which patients would benefit from each treatment [25].

In the present study, we investigate how treatment efficacy can be optimized by tailoring the alliance function to the patients’ attachment orientation. The primary aim of this RCT is to examine prospectively the differential efficacy of supportive and supportive-expressive treatment for patients with different attachment orientations. We propose that counter-complementary techniques can challenge the patients’ tendencies in interpersonal relationships and encourage corrective interpersonal experiences [19, 38], resulting in better treatment outcome. Our primary hypothesis is that attachment orientation has a significant moderating effect on treatment approach in predicting outcome. Specifically, we hypothesize that anxiously attached patients benefit most from supportive-expressive treatment, where the trait-like component of the alliance plays a dominant role, whereas avoidantly attached patients benefit most from supportive treatment, where alliance functions as an active ingredient in itself. Our secondary hypothesis is that SE shows better outcome than supportive treatment at the one-year follow-up [39]. After testing the main hypotheses, we will examine other processes and mechanisms of change, clinical predictors, perceptions of change by both patients and therapists as well as the agreement between them, implicit measures, biomarkers of therapeutic change, acoustic markers, and motion synchronicity, among others, in several consecutive studies.

## Method

### Study design

The study is designed to reach an assignment ratio of 1:1 for supportive-expressive vs. supportive treatment. Assignment to treatment arm is conducted by an outside institution, not involved in the study, the Biostatistics Department of the Gertner Institute for Epidemiology and Health Policy Research. Assignment to treatment arm is based on the minimization algorithm [40]). Factors for balancing are age (higher or equal vs. lower than 30), gender (male vs. female), family status (married/cohabiting vs. not married/cohabiting), baseline 17-item Hamilton Rating Scale, HRSD [41] (higher or equal vs. lower than 20), baseline attachment avoidance (higher or equal vs. lower than 3.5 on the Experience in Close Relationships, ECR [31]; see details in the Measures section), baseline attachment anxiety (higher or equal vs. lower than 3.5 on the ECR), and personality disorders (present vs. absent). Except for the therapist, the entire research team is blind to the patients’ assignment to treatment.

### Participants

One hundred patients with MDD are being recruited through advertisements in the central regions of Israel, offering free treatment at the psychotherapy research lab clinic.

#### Inclusion criteria

(a) MDD diagnostic criteria using the structured clinical interviews for DSM-V, with scores above 14 on the 17-item HRSD at two evaluations, one week apart [41], and current MDD based on the MINI (International Neuropsychiatric Interview; [42]); (b) if on medication, patients’ dosage must be stable for at least three months before the start of the study, and they must be willing to maintain stable dosage for the duration of treatment; (c) age between 18 and 60 years [43]; (d) Hebrew language fluency; (e) provision of written informed consent.

#### Exclusion criteria

(a) current risk of suicide or self-harm (HRSD suicide item > 2); (b) current substance abuse disorder; (c) current or past schizophrenia or psychosis, bipolar disorder, or severe eating disorder, requiring medical monitoring; (d) history of organic mental disease; (e) currently in psychotherapy.

### Treatments

Patients will receive 16 50-min sessions of SE treatment (SE; [17, 18]), a time-limited psychodynamic therapy adapted for depression, either in an expressive-focused condition (including the use of expressive techniques, such as interpretation, confrontation, clarification), or in a supportive-focused condition (including the use of supportive techniques, such as affirmation and empathic validation). We use comprehensive treatment protocols. For SE treatment, we use the Luborsky [17, 18] manualized treatment. The supportive condition includes all supportive techniques detailed in the manual used by Luborsky [17, 18], but forbids the use of any expressive techniques, as detailed in [20]. After the end of treatment, once a month, a maintenance session will be provided to all patients with their treating therapists, for a total of 4 follow-up sessions.

### Therapists

Therapists will act as their own controls to avoid nesting of therapists within treatment conditions, which may result in unwanted confounding. This approach has been found to be feasible in previous trials [44]. To control for therapist allegiance, all therapists will be asked to report their preferences before the start of treatment, and we will control for these preferences in the analyses [45].

Therapists interested in participating in the RCT answered ads seeking psychologists with at least five years of expertise in psychodynamic treatment, willing to participate in an RCT on short-term psychodynamic treatment. After interviews, eight therapists were accepted into the study. All had formal training and experience in psychodynamic treatment. The therapists attended a 20-h training workshop in supportive and expressive techniques. The training included formal teaching and role playing, using the different techniques. Two of the therapists did not continue after the training phase for various reasons (one being offered a full-time position elsewhere, the other demonstrating low levels of adherence).

All therapists completed treatment of two pilot patients, one of each treatment type, and had to demonstrate sufficient adherence before moving into the trial phase. During the pilot phase, and after the start of the research, each therapist received weekly group supervision from two supervisors, as well as weekly individual supervision from one of the supervisors. Individual and group supervisions make extensive use of videotaped sessions for feedback. The supervisors are licensed clinical psychologists, with extensive supervision experience. The supervisors receive supervision concerning the supervision process from an international expert in SE, with more than 20 years of experience in psychodynamic treatment for depression, and more than 15 years of experience in SE treatments in RCTs.

### Fidelity check

We use the Penn Adherence-Competence Scale (PACS; [46]) to examine the degree to which therapists adhere to the manual in the techniques they are using, whether they are competent in the use of the technique, and whether they avoid the use of techniques that are forbidden (based on the condition to which their patient was assigned). The PACS includes 3 subscales: general therapeutic behaviors, the supportive component, and the expressive component [46]. The two conditions of the present study are expected to differ on the expressive component. The research team is supervised by an international expert on the use of the PACS, with vast experience in using the PACS in RCTs on SE treatment. In instances of low adherence or competence, relevant supervision tools will be deployed (e.g., [47]) [48]. As detailed above, therapists attend weekly individual and group supervisions for the entire duration of their participation in the study. In the supervision session, videotaped sessions are being analyzed, looking for instances of deviations from the manual to be worked through.

### Procedure and randomization

**Fig. 1:**
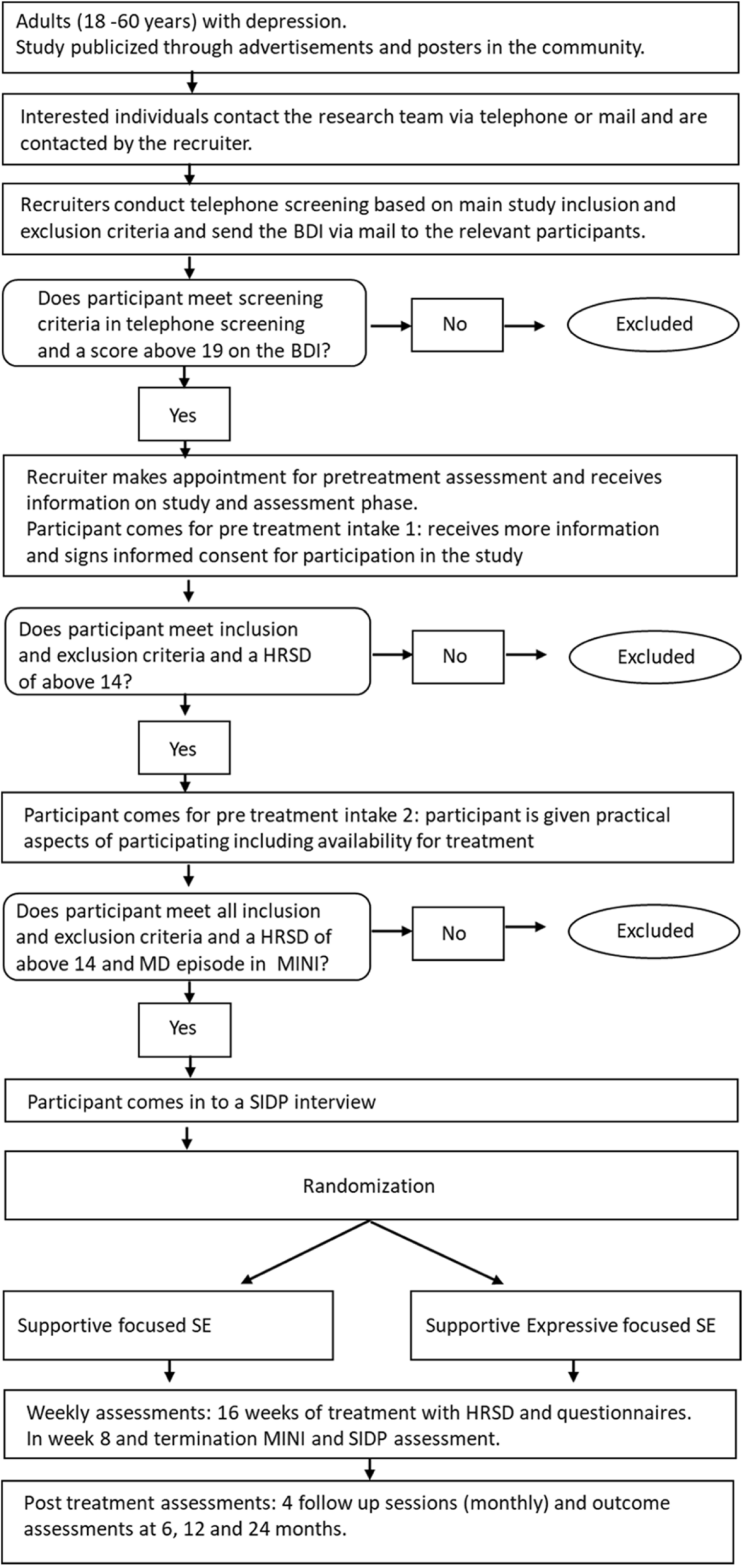
Flow of participants in the study

Applicants are recruited by self-referral, based on advertisements (Fig. 1). Recruitment information is disseminated by posters, local media, and online publications (social media, etc.). Application is by email or phone. In an initial phone screening, the interested individuals are informed about the study procedure and the relevant inclusion and exclusion criteria. If there are indications of depressive complaints, applicants who consent to the research intake procedure are asked to complete the BDI-II (Beck Depression Inventory; [49]). To ensure high levels of severity of depressive symptoms, applicants must score at least 19 points on the BDI-II. Applicants who scored 19 points and above on the BDI-II are contacted to schedule an initial intake meeting during which researchers explain the study, and there is opportunity to discuss any questions. Applicants receive written and oral information about the content and extent of the planned study. This includes information about the treatment, including safety and possible risks, the information that all treatment sessions are videotaped, and about their right to withdraw from the research at any time. Applicants who agree to participate are required to sign the informed consent form. At the first face-to-face assessment, the HRSD interview is administered, and participants receive a baseline battery of questionnaires, including demographic information, concurrent medication, and measures of personality characteristics and comorbid conditions. At the second interview, the HRSD and the MINI [42] are administered to measure the presence and severity of baseline symptom and comorbid conditions, and participants provide saliva samples.

The first and the second interviews of all participants, as well as all the assessments before and after each session, are administered by the same evaluator who has been extensively trained and was found to be reliable in the use of the HRSD and the MINI. The evaluators are trained in the administration of both measures. After successfully completing two months of extensive training and achieving high reliability, evaluators begin by observing another evaluator at work, after which they administer the measures with a trained evaluator for several weeks, before performing the evaluation by themselves. Throughout the trial period, the reliability of the evaluators is evaluated on a weekly basis.

After the second interview, a third face-to-face meeting is scheduled, at which the Structured Interview for DSM-IV Personality, SIDP [50] is administered by a different trained and reliable evaluator, to assess comorbid personality disorders. The evaluators are trained in the administration of the measure. After successfully completing extensive training and achieving high reliability, evaluators begin by observing another evaluator at work. Next, they administer the measures together with a trained evaluator for several weeks, before performing the evaluation by themselves. Throughout the trial period, the reliability of the evaluators is assessed every week. If eligible for the study, participants receive further written and oral information about the treatments and about the full research procedure and assessments, before being randomized to one of the treatment conditions. They also indicate whether they can be contacted again after the study for additional waves and for extending the follow-up period beyond the specified two years.

Applicants who are not eligible for the study (having not met the inclusion criteria or having met an exclusion criterion), yet request psychotherapy, are referred to appropriate care. Participants meeting inclusion criteria are randomized to one of the two treatment arms. Researchers, assessors, intakers, and the entire research team remain blind to treatment allocation. The only team members who are exposed to treatment assignment are, by necessity, the therapists and clinical supervisors. The following strategies are used to minimize bias that could arise from revealing treatment allocation: (a) all patients are blind to treatment allocation; (b) all process and outcome assessors are blind to treatment allocation; (c) therapists are regularly reminded that they are not allowed to disclose treatment allocation to any member of the team; (d) patients and outcome assessors are asked to guess which treatment was given so that the effects of possible bias can be examined in the analysis; and (e) all outcome assessor interviews are audiotaped, and 30% randomly assigned tapes rerated by independent rates. If blindness is broken, all subsequent assessments will be carried out by an alternative assessor. If patients withdraw from treatment before the end of active treatment, dropout questionnaires are administered to both the patient and the therapist (Post Session Questionnaire, PSQ; [51]). In the course of the active phase of treatment, the HRSD is measured weekly, and self-report questionnaires are administered. Saliva samples and cognitive tasks are measured before the start of treatment and on weeks 4, 8, 12, and 16. The MINI and SIDP are measured at weeks 8 and 16.

After the completion of the active phase (16 weeks), the first four follow-up sessions include maintenance sessions with the therapist, HRSD administration, and completion of a battery of self-report questionnaires. The MINI is administered at the first and third follow-up meetings. The three subsequent follow-up sessions do not include sessions with the therapist, and are scheduled for two months after the last follow-up session (six months after the end of the active treatment phase of 16-weeks), one year after the end of the active phase, and two years after the end of the active phase. At these three additional follow-up sessions, the HRSD and the MINI clinical interviews are administered, and self-report questionnaires are completed. In the first two additional follow-up sessions the SIDP clinical interviews is administered as well.

### Measures

The primary outcome measure of the trial is the HRSD [41], a 17-item clinically administered measure assessing the severity of depression. The assigned score is calculated by summing up all 17 items. Using, among others, the measures listed below, we also assess secondary outcomes, such as self-reported symptoms (i.e., BDI; [49]; BAI, Beck Anxiety Inventory; [52], OQ-30; [27]), interpersonal functioning (i.e., IIP, Interpersonal Problems Inventory; [53, 54], ECR, Experience in Close Relationships; [31]), and quality of life (Q-LES-Q, Quality of Life Enjoyment and Satisfaction; [55]). We also assess several process measures, such as the working alliance (WAI, Working Alliance Inventory; [56]) and therapeutic interventions (MULTI, Multi-theoretical List of Therapeutic Interventions; [57]).

### Data analyses

All analyses will follow the intention-to-treat principle. Characteristics of the treatment groups will be described at baseline. To examine the potential moderating influence of attachment anxiety and attachment avoidance on the effect of treatment condition on outcome, we will use a multilevel hierarchical linear model with observations nested within patients. We will test two 3-way interactions of time, treatment condition, and attachment orientation (anxiety, avoidance) in predicting HRSD during active treatment (until Week 16).

### Ethical considerations

The study design, procedure, and informed consent form were approved by the University of Haifa ethical committee (approval number: 118/15, Date: 10/10/2015). Participants receive detailed information about the study procedure in an oral explanation and in writing. Participants receive complete information regarding the implications of their participation, including potential risks, inconvenience, and benefits, their ability to stop their participation at any time without adverse consequences, issues of confidentiality, etc. An elaborate data management plan ensures careful handling of confidential data in all stages of the research process. In case of a serious adverse event (e.g., critical suicide risk), the RISK protocol will be activated, and a special committee trained to handle such situations, including three licensed clinical psychologists, will immediately take charge.

## Discussion

MDD is the leading cause of disability worldwide [58]. Several treatments for MDD have been found to be similarly effective and more effective than control conditions, including supportive-expressive and supportive treatment [21]. Yet, about half the patients receiving any treatment for MDD fail to show adequate response. Although there is low variance between distinct psychotherapies for MDD, there is high variance within each psychotherapy condition. Secondary post hoc analyses on RCT datasets suggest that different subgroups of MDD patient respond best to different treatments, highlighting the need for progress toward personalized treatment for MDD [22, 25]. Currently, there are few evidence-based tools to guide clinicians in patient assignment to their optimal treatment.

The present RCT was designed to test an a priori hypothesis regarding the best treatment for patients with distinct attachment orientations, based on the accumulating literature regarding two distinct roles that alliance can play in treatment, specifically in supportive vs. supportive-expressive treatment. We expect to make important progress in uncovering who may benefit most from each treatment, capitalizing on distinct roles of alliance. This information can then be used in future trials to assign patients to their optimal expected treatment vs. the usual allocation in clinical practice.

## Abbreviations

BAI: Beck anxiety inventory
BDI: Beck depression inventory
CCRT: Core conflictual relationship themes
ECR: Experience in close relationships
HRSD: Hamilton rating scale for depression
IIP: Inventory of interpersonal problems
MDD: Major depressive disorder
MULTI: Multi-theoretical list of therapeutic interventions
OQ: Outcome questionnaire
PACS: Penn adherence-competence scale
PSQ: Post-session questionnaire
Q-LES-Q: Quality of life enjoyment and satisfaction
RCT: Randomized controlled trial
SE: Supportive-expressive
SIDP: Structured interview for DSM-IV personality
WAI: Working alliance inventory
